# Histological Processes in Divided Nerves

**Published:** 1873-02

**Authors:** 


### ARTICLE X.

### *Histological Processes in Divided Nerves.*

Under the title of "Histological Processes in Divided Nerves," Dr. Benecke, of Konigsberg, contributes an important paper to the last part of Virchow's Archiv. His investigations, he says, have extended over many years, and he has experimented upon cats, rabbits, crows, fowls, pigeons, and various small birds. Of all these he finds young cats the best adapted for research, as the nerve trunks in those animals contain very little connective tissue in their interior. The mode of operating consisted either in dividing the nerve with the knife, or in applying a fine silk ligature for a few moments, which is almost equally efficacious in cutting through the axis cylinders. The changes induced were examined at various intervals of time, ranging from two hours to six months, and both in the fresh state and after maceration in different preserving fluids. The first changes observed, and these take place within a few hours after section, are that the two extremities of the nerve adjoining the cut become of a greyish or yellowish-red color, cloudy aspect, and more or less strongly swollen. These changes are due to congestion of the severed blood vessels, and to the imbibition of the serous transudate. In a short time the part of the centric extremity lying near the cut, and the whole peripheric part assumes a cloudy yellow tint, becomes somewhat attenuated, and tears easily. The sheath of Schwann undergoes, at first at least, very little alteration; the medullary sheath, on the other hand, coagulates, and breaks down into a finely granular detritus. The axis cylinder early appears enlarged and swollen, but soon attenuates, and ultimately disappears entirely. In the later stages of the process of disintegration it is impossible by any means to bring it into view. Benecke has never been able to find that the degenerative process progresses in an orderly manner in either direction from the lesion, but believes that it takes place *coincidently* throughout the whole length of the peripheral extremity, and in the immediately adjoining portion of the centric end.

But even whilst disintegration is advancing, the process of regeneration has commenced in the form of multiplication by fission of the nuclei of the neurilemma, or sheath of Schwann. These soon become converted into elongated fusiform bodies, and after a time form the only contents of the empty and collapsed sheaths, which then, he thinks, disappear. The protoplasmic processes of the new elongated nuclei now undergo fusion, and unite to form pale, slender bands coincidently in the cicatrix and in the peripheral end of the divided nerve, which is thus brought into organic connexion with the central stump. In the further progress of the regenerative process a medullary sheath is gradually formed around the primitive bands or cylinder axes originating from the nuclei. These last no longer retain their ordinary appearance, but become homogeneous—a few only remaining in each fibre, from which the normal sheaths of Schwann are afterwards developed.

Upon the whole, therefore, it would appear that after section the proper nervous tissue of the entire peripherical portion of the nerve undergoes disintegration and disappears, whilst regeneration is effected by a process precisely analogous to that which many observers have demonstrated to occur in the natural development of the nerves in the tail of the tadpole, and in the embryo of the fowl; the cylinder axis being first formed by the coalescence of nuclei, to which the white or medullary substance and the sheath of Schwann are subsequent additions.—*London Lancet.*

